# Supplementary figures and images for: Eigen-Epistasis for detecting gene-gene interactions
Source: BMC Bioinformatics. 2017 Jan 23;18:54. doi: 10.1186/s12859-017-1488-0 (PMC5259960; doi:10.1186/s12859-017-1488-0)

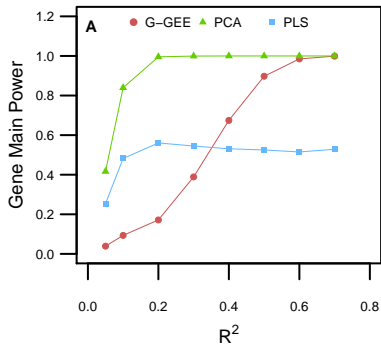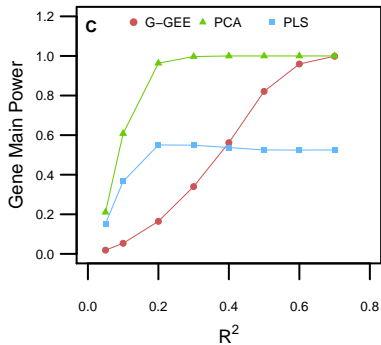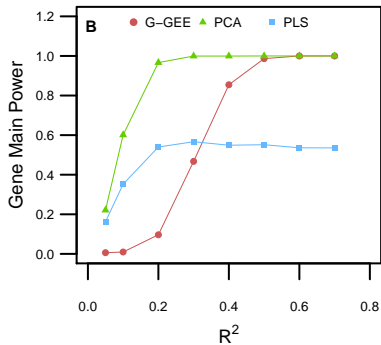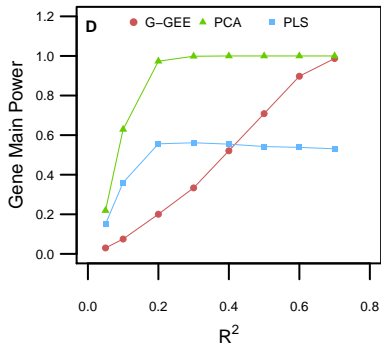

Supplement: Additional file 1 — Figure S1. Comparison of power to detect main effects in the simplified simulation study. Power under a simplified context. The figures show the power to detect main effects of the three methods depending on R 2. (PDF 8 kb) [file 12859_2017_1488_MOESM1_ESM.pdf]

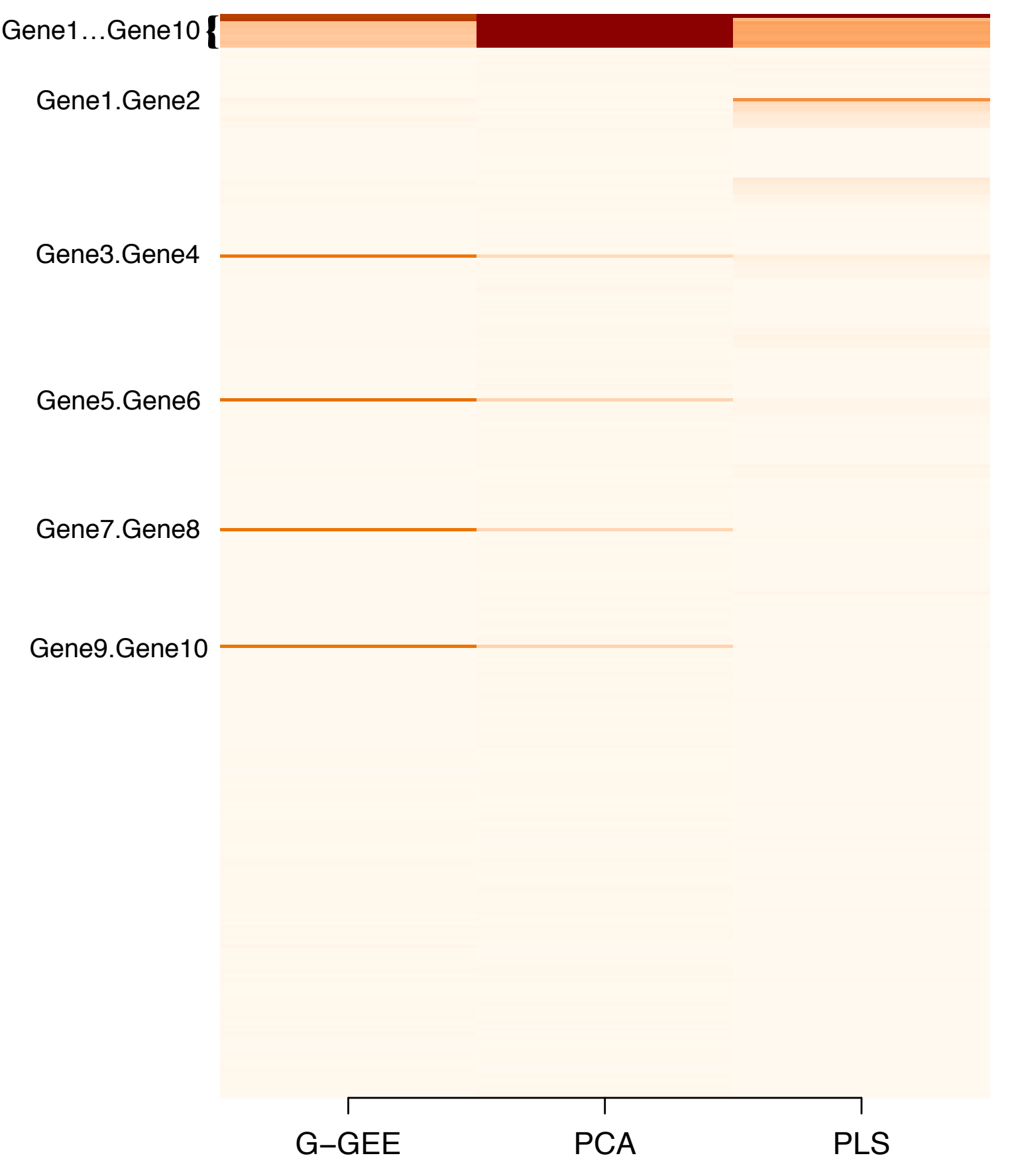

Supplement: Additional file 2 — Figure S2. Simulation on 25 genes with fully simulated data and various simulated effects. Discoveries for the setting with numerous effects. Heatmap of the ratio of the number of times where each variable was significant to the total number of simulations for R 2=0.7 using the Wang Pathway model for the phenotype simulation with fully simulated data. We consider 25 genes with two main effects for genes 1 and 2, and four interaction effects between genes 3 and 4, genes 5 and 6, genes 7 and 8, and genes 9 and 10. (PDF 30 kb) [file 12859_2017_1488_MOESM2_ESM.pdf]

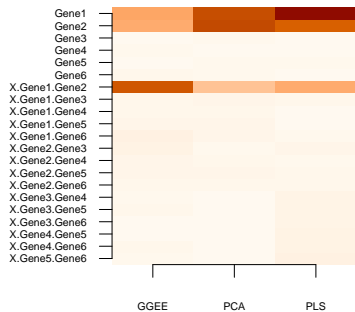

Cadre A ;  $R^2=0.4$

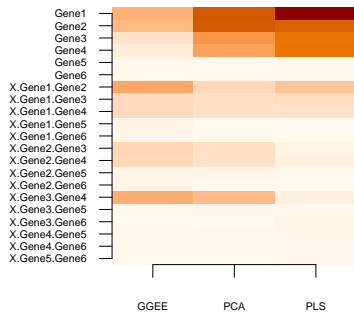

Cadre C ;  $R^2=0.4$

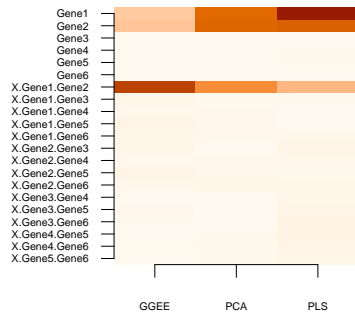

Cadre E ;  $R^2=0.4$

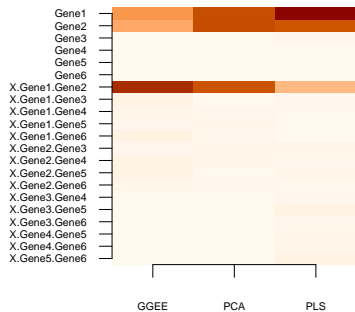

Cadre B ;  $R^2=0.4$

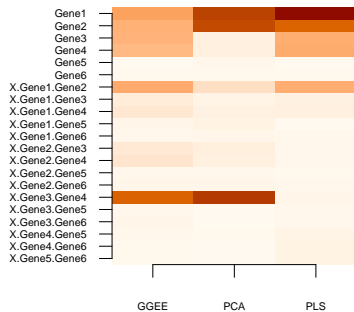

Cadre D ;  $R^2=0.4$

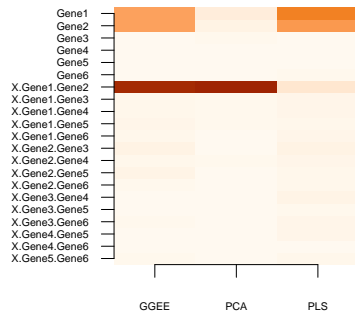

Cadre F ;  $R^2=0.4$

Supplement: Additional file 3 — Figure S3. Discoveries in the realistic simulation study with R 2=0.4. Discoveries under a realistic context. Heatmaps of the ratio of the number of times where each variable was significant to the total number of simulations for R 2=0.4. (PDF 9 kb) [file 12859_2017_1488_MOESM3_ESM.pdf]

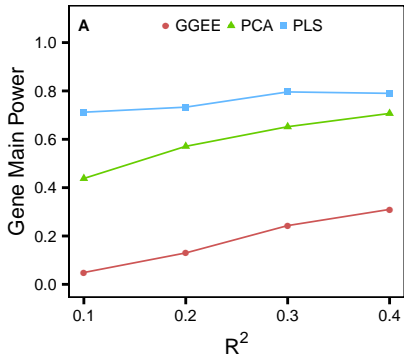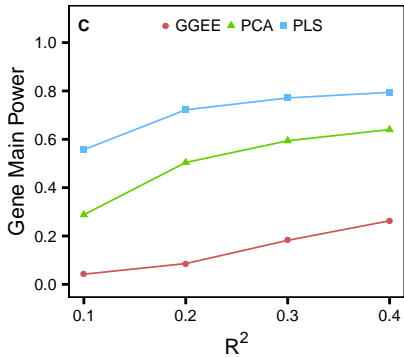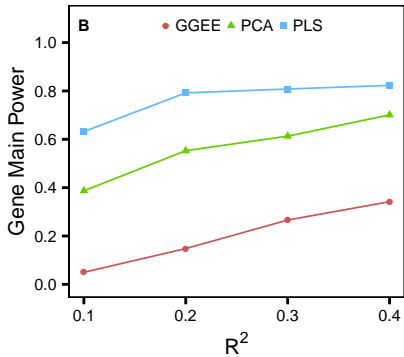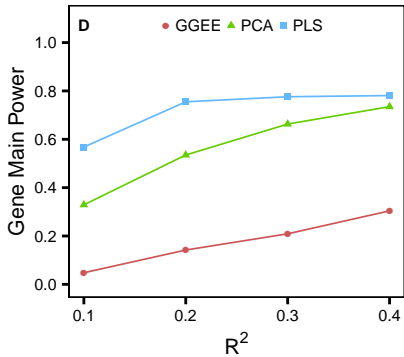

Supplement: Additional file 5 — Figure S5. Comparison of power to detect main effects in the realistic simulation study. Power under a realistic context. The figures show the power to detect main effects of the three methods depending on R 2. (PDF 7 kb) [file 12859_2017_1488_MOESM5_ESM.pdf]
