# Supplementary material for: Eigen-Epistasis for detecting gene-gene interactions
Source: BMC Bioinformatics. 2017 Jan 23;18:54. doi: 10.1186/s12859-017-1488-0 (PMC5259960; doi:10.1186/s12859-017-1488-0)
Supplement: Additional file 4 — Figure S4. Comparison of execution time required to model interaction and to fit Group Lasso for the five first settings of the realistic simulation study. Execution time. Median of the execution time to model interaction and to fit Group Lasso for the five first settings of the realistic simulation study. (PDF 8 kb) [file 12859_2017_1488_MOESM4_ESM.pdf]

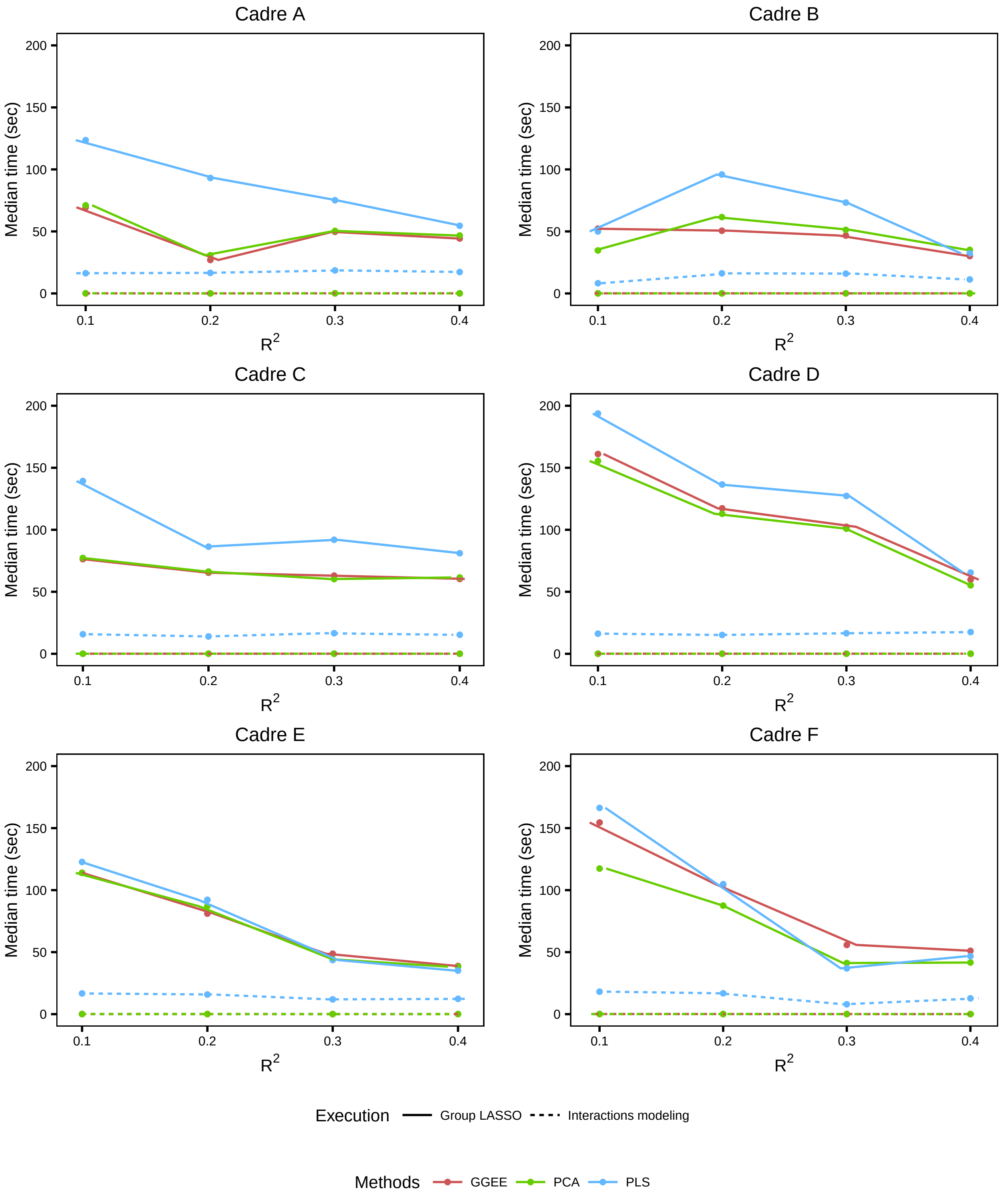

Cadre A
Cadre B
Cadre C
Cadre D
Cadre E
Cadre F
Median time (sec)
0
50
100
150
200
0.1
0.2
0.3
0.4
R^2
Execution
Group LASSO
Interactions modeling
Methods
GGEE
PCA
PLS
